# Unlocking the Memory Component of Alzheimer’s Disease: Biological Processes and Pathways across Brain Regions

**DOI:** 10.3390/biom12020263

**Published:** 2022-02-06

**Authors:** Nikolas Dovrolis, Maria Nikou, Alexandra Gkrouzoudi, Nikolaos Dimitriadis, Ioanna Maroulakou

**Affiliations:** 1 Laboratory of Pharmacology, Department of Medicine, Democritus University of Thrace, 68100 Alexandroupolis, Greece; ndovroli@med.duth.gr; 2 Laboratory of Genetics & Genomics of Cancer and Chronic Diseases, Department of Molecular Biology & Genetics, Democritus University of Thrace, 68100 Alexandroupolis, Greece; manikou@affil.duth.gr (M.N.); agkrouzo@affil.duth.gr (A.G.); nikolaosdimitriathis@gmail.com (N.D.)

**Keywords:** Alzheimer’s Disease, memory, computational, co-expression, functional analysis

## Abstract

Alzheimer’s Disease (AD) is a neurodegenerative disorder characterized by a progressive loss of memory and a general cognitive decline leading to dementia. AD is characterized by changes in the behavior of the genome and can be traced across multiple brain regions and cell types. It is mainly associated with β-amyloid deposits and tau protein misfolding, leading to neurofibrillary tangles. In recent years, however, research has shown that there is a high complexity of mechanisms involved in AD neurophysiology and functional decline enabling its diverse presentation and allowing more questions to arise. In this study, we present a computational approach to facilitate brain region-specific analysis of genes and biological processes involved in the memory process in AD. Utilizing current genetic knowledge we provide a gene set of 265 memory-associated genes in AD, combinations of which can be found co-expressed in 11 different brain regions along with their functional role. The identified genes participate in a spectrum of biological processes ranging from structural and neuronal communication to epigenetic alterations and immune system responses. These findings provide new insights into the molecular background of AD and can be used to bridge the genotype–phenotype gap and allow for new therapeutic hypotheses.

## 1. Introduction

When Alois Alzheimer was first introduced to Auguste Deter, more than 100 years ago, he faced a strange dementia which presented in a non-geriatric case. Although the symptomology of behavioral changes and memory loss was not atypical for what the scientific society had previously observed, his histological experiments on the brain of the then deceased patient were unique and provided the basis of what we now know to be tau-associated neurofibrillary tangles (NFTs) and β-amyloid (Aβ) plaques in what is known as Alzheimer’s Disease (AD) [1]. AD is considered a typical neurodegenerative disease with high prevalence in older individuals, a relatively stable progression akin to other similar disorders like Parkinson’s Disease, exhibiting some molecular commonalities but different symptomology and etiopathology [2].

Regardless of different etiopathology and histological presentations, dementias share the characteristic of memory impairment. Short- or long-term, linked to sensory stimulation or emotion, memory function is important for our life, communication, and even more complex operations like learning [3]. The way memory is affected by disease progression has been the target of AD research and has provided information on symptomatic onset and progression through different brain regions. The hippocampus and the amygdala are two brain regions among the first to show histological abnormalities and thus are significant in understanding memory deterioration. The hippocampus allows for the formation of new memories via experience and stimuli [4,5] and is responsible for the consolidation of new information via short to long-term spatial memory transition [6]. Severe damage to the hippocampus can lead to, among other symptoms, disorientation [7], verbal memory retention [8] and difficulty encoding emotional responses from the amygdala which impairs episodic memory [9]. Regarding the amygdala, it appears that it synergizes with many brain regions, not just the hippocampus, to modulate memory consolidation [10] but also is responsible for emotional memory confinement [11,12]. Following the hippocampus and amygdala, another member of the limbic system, the hypothalamus, displays deterioration in later stages of AD progression, but is also heavily involved in the memory process affecting memory updating [13] and the short-term working memory [14]. Traversing the brain pathology during AD brings forth a complex of cortices, including the frontal, the cerebral and the anterior cingulate cortex, responsible for storing mainly short-term memories [15,16,17] and working closely with the limbic system for emotional memory processing [18]. The basal ganglia and its members, the putamen, the caudate nucleus and the nucleus accumbens, have also shown involvement in memory enhancement [19] and general memory function [20,21]. Other parts of the brain such as the substantia nigra and the cerebellum are not traditionally linked to AD, with both having serious implications in the motor function of other degenerative disorders, such as Parkinson’s Disease, but there are indications that specific memory functions are facilitated by them [22,23].

Modern approaches to identifying the role of specific brain regions in AD pathophysiology employ advanced functional connectivity approaches. Using imaging and testing techniques like fMRI [24,25] and EEG [26,27] researchers have been able to identify key interactions between brain regions which apply to specific individuals leading to a more personalized approach. By deciphering the crosstalk networks formed by the data obtained through these methodologies, studies have been able to improve on the more traditional atlas-based approaches [28,29] which have been used to identify mild cognitive impairment in Alzheimer. All these approaches are based on observations made in the functionality of the brain and the signals these produce, or rather fail to produce, during AD, but offer little information on the molecular basis of why that is happening. Although very important in diagnosing AD and memory impairment, functional connectivity approaches are better suited to see the disease’s progression/damage in real time and to better understand its effects.

Thus, in this work, by leveraging current knowledge of genes associated with memory from various neurodegenerative disorders and genes associated with AD in general, we highlight the molecular background of the mechanisms of memory in different brain regions during AD. We hope that this will provide the basis for further studies on how the molecular background of memory impairment in AD can be used to understand and possibly be targeted by therapeutic approaches.

## 2. Methodology

### 2.1. Literature-Based Gene Retrieval

At first, two different gene sets were created. Gene Set A was comprised of genes which are known to play a role in memory development, maintenance and impairment. The data were taken from DisGeNET [30], Ensembl genome browser [31], OpenTargets [32], GWAS catalog of EMBL-EBI [33] and PubMed published works by using the following search terms: “memory”, “memory impairment”, “memory loss”, “memory dysfunction”, “age related memory disorders”, “memory performance”, “poor short term memory”, “working memory”, “episodic memory”, “forgetful”, “amnesia”, “temporary amnesia”, “verbal memory”, “visual memory”, “sensory memory”, “emotional memory”. Gene Set B contains all the genes known in DisGeNET as being associated with AD related terms, namely “Alzheimer’s Disease”, “Alzheimer Disease, Late Onset”, “Alzheimer Disease, Early Onset”, “ALZHEIMER DISEASE, FAMILIAL, 1”, “Alzheimer disease, familial, type 3”, “Familial Alzheimer Disease (FAD)”, “Alzheimer’s Disease, Focal Onset”, “Prodromal Alzheimer’s disease”, “Alzheimer disease type 1”, “ALZHEIMER DISEASE 5”, “Alzheimer Disease 12”, “ALZHEIMER DISEASE 2”, “Alzheimer Disease, Familial, 3, with Spastic Paraparesis and Unusual Plaques”, “Tauopathies”, “trisomy [21]”.

We propose that the intersection of these 2 gene sets allows one to identify which of the genes, already highlighted in literature as involved in AD, play an important role to its characteristic memory deterioration. These common genes were identified using a Venn diagram produced by VENNY [34] and comprise a new gene set, henceforth referred to as the Memory in AD gene set (MADgs), to be used in all further analyses.

### 2.2. Brain Region Co-Expression

To identify the role of the MADgs genes in specific brain regions and how those are involved in AD, the online platform NetworkAnalyst [35] and its co-expression by tissue function was used. This specific function leverages knowledge from the iNetModels 2.0 database [36] to highlight genes which were found to be co-expressed in a multitude of experimental data from various tissues. We argue that a higher number of genes from MADgs found to be co-expressed per brain region (seeds as they are referred to on the platform) signifies a stronger significance of the specific tissue in elucidating their involvement in AD. In total, 11 regions were investigated: the amygdala, the anterior cingulate cortex, the caudate basal ganglia, the cerebellum, the cortex, the frontal cortex, the hippocampus, the hypothalamus, the nucleus accumbens, the putamen, and finally the substantia nigra. To find the overlap of genes among these regions, list of intersection tools from the webpage molbiotools [37] and the VENNY platform were used.

### 2.3. Protein–Protein Interaction Network

Admittedly the biological background of any disease phenotype is more complex than any simple gene list identified by various omics approaches. For this reason, we used the MADgs as input for a protein–protein interaction network analysis through NetworkAnalyst and its STRINGdb [38] API using a confidence score cut-off of 900. This allowed for a broader enrichment of the MADgs along with the ability to identify biologically significant proteins using network topology metrics such as node degree and betweenness (hub and bottleneck nodes, respectively). STRINGdb is based on protein interactions found in literature and experiment databases.

### 2.4. Functional Analysis

Functional analysis was performed on the MADgs as a whole, but also on the seeds per brain region, using two popular pathway and gene signaling databases, Reactome [39] and Gene Ontology Biological Processes (GO-BP) [40]. All our findings were manually clustered in broader categories which include multiple pathways and compared among the brain regions involved in AD.

### 2.5. miRNA Analysis

Finally, we used the MADgs in its entirety to identify miRNAs which have verified interactions with our genes. The R package multimir [41] produced mature miRNA lists for each one of the genes in MADgs by querying miRNA-target interactions on 3 databases: mirecords [42], mirtarbase [43], and tarbase [44].

**Figure 1:**
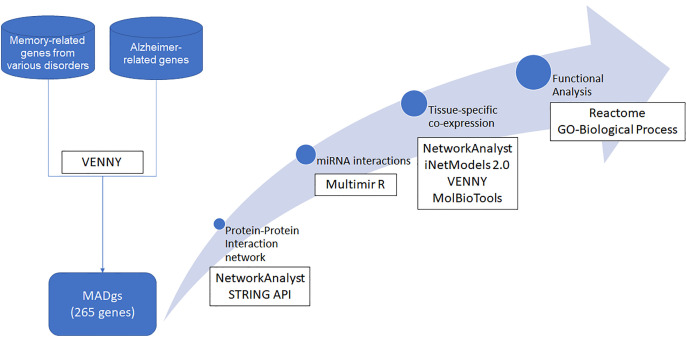
Visual representation of the methodological steps taken. Rectangles with a white background encase the tools utilized.

The entire methodological approach is summarized in Figure 1.

## 3. Results

### 3.1. Genetic Background of Memory in AD

**Figure 2:**
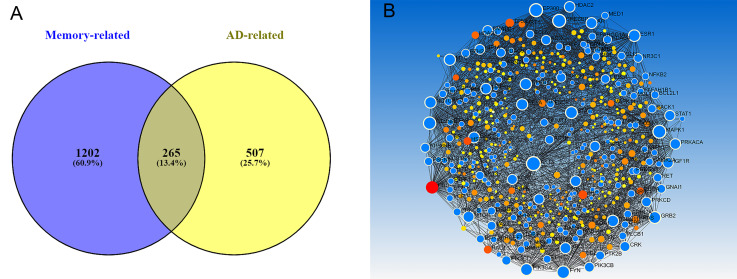
(**A**) Venn diagram representing the intersection between the memory related genes from various neurodegenerative disorders and the general Alzheimer’s Disease genes. Thei cross-section is called the Memory in Alzheimer’s Disease gene set (MADgs). (**B**) Protein–Protein Interaction network produced by the MADgs (blue nodes = MADgs genes).

**Figure 3:**
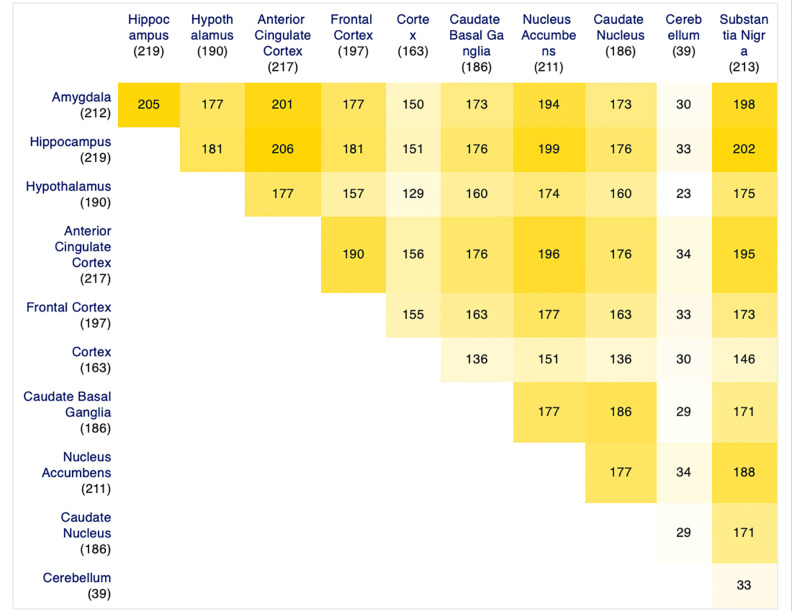
Intersections of the MADgs genes found co-expressed in 11 brain regions.

**Figure 4:**
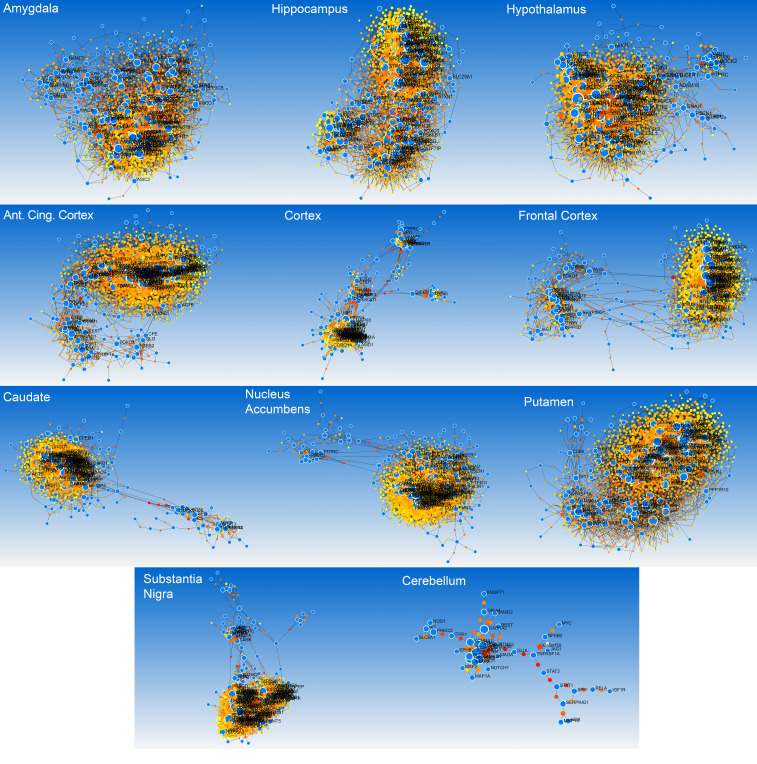
Co-expression networks of the MADgs genes in 11 different brain regions. Blue nodes are our input genes from MADgs (seeds).

As previously described, we reviewed several sources to identify both genes associated with memory development and impairment, but also with AD itself. Gene Set A (the memory associated genes from a variety of disorders) was comprised of a total 2743 genes. Gene Set B (the AD associated genes) contained a list of 1014 genes. Their intersection, (Figure 2A) referred to as MADgs, includes 265 genes used for our analyses. All three lists can be found in Supplementary File S1. Although there were a lot of known prominent AD genes highlighted in MADgs, their volume warranted further investigation. The protein–protein interaction network (Figure 2B) created from the MADgs and enriched, as previously described, helped evaluate the gene list further. In descending order, the top 10 hub nodes (highest degree centrality) were *AKT1*, *UBC*, *EP300*, *CREBBP*, *MAPK1*, *FYN*, *CTNNB1*, *PIK3CA*, *GRB2* and *STAT3*, whereas the top bottleneck nodes (highest betweenness centrality) were *UBC*, *AKT1*, *CTNNB1*, *FYN*, *EP300*, *MAPK1*, *CREBBP*, *PRKACA*, *GRB2* and *NOTCH1.* Interestingly, all these genes were part of MADgs, except for UBC (Ubiquitin-C) which appears to be quite significant for our biological network and it is understudied for its role in AD in the current literature. Wanting to focus on which of those genes play an active role in specific brain regions we employed the co-expression network approach. Figure 3 summarizes the number of genes found co-expressed per brain tissue, but also their overlap, whereas Figure 4 depicts their enriched co-expression networks. The amygdala and the hippocampus have the highest overlap with other brain tissues regarding genes of MADgs co-expressed, whereas the cerebellum shows minimal overlap.

**Figure 5:**
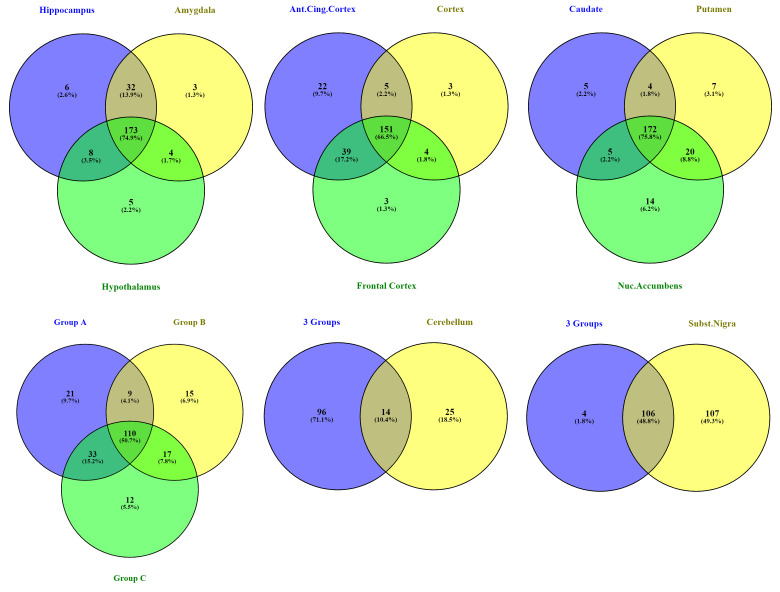
Venn diagrams representing the intersection of co-expressed genes in different brain regions and combinations of them.

As shown in the introduction, AD has a known course of pathology in brain tissue and thus we wanted to extract some more information based on that knowledge. The brain regions for which we had co-expression networks were divided into subgroups. The first one (Group A) includes the hippocampus, the amygdala and the hypothalamus which are known to be the first affected regions and are part of the limbic system, the second (Group B) includes the cortex, the frontal cortex and the anterior cingulate cortex, the third (Group C) includes the putamen, caudate nucleus and nucleus accumbens, which all partially compose the basal ganglia. Finally, the substantia nigra and the cerebellum were investigated separately since the former is more prominent for its involvement in Parkinson’s Disease (and also considered to be involved mainly in late-stage AD) and the latter showed minimal involvement of memory related genes in our co-expression analysis. Figure 5 presents the gene overlap among the members of the first three groups, respectively, the overlap among the whole groups and how the latter intersects with substantia nigra and the cerebellum. The entire list of genes for each Venn of Figure 5 can be found in Supplementary File S2. To summarize these results, Groups A and C have the largest overlap among their members with 173 and 172 out of the 265 genes of MADgs, respectively, whereas Group B has 151 genes intersecting among its members. In total, 110 genes are commonly co-expressed in the nine brain regions of these three groups and only 14 of them appear to be intersecting with the cerebellum (which had only a few MADgs genes co-expressed to start with) while 104 are common in their intersection with the substantia nigra.

Since the genetic background of any disorder can also be affected by the involvement of the small miRNAs our investigation was expanded to elucidate their role as well. In total, 2219 miRNAs were found to interact with at least 1 of the genes in MADgs, and of those 66 interact with at least 50 genes. There are also some small clusters of miRNA families which can be extracted from this list, such as the let-7 family which includes 7/66 miRNAs. The top 10 miRNAs are *hsa-miR-16-5p*, *hsa-miR-124-3p*, *hsa-miR-27a-3p*, *hsa-miR-34a-5p*, *hsa-miR-155-5p*, *hsa-miR-1-3p*, *hsa-let-7b-5p*, *hsa-miR-107*, *hsa-miR-7-5p* and *hsa-miR-128-3p* which interact with 145, 135, 129, 121, 121, 113, 102, 97, 96 and 95 genes from MADgs, respectively. The entire table of miRNAs, their families, which individual genes from MADgs they affect, and their total number of interactions can be found in Supplementary File S3.

### 3.2. Functional Background of Memory in AD

**Figure 6:**
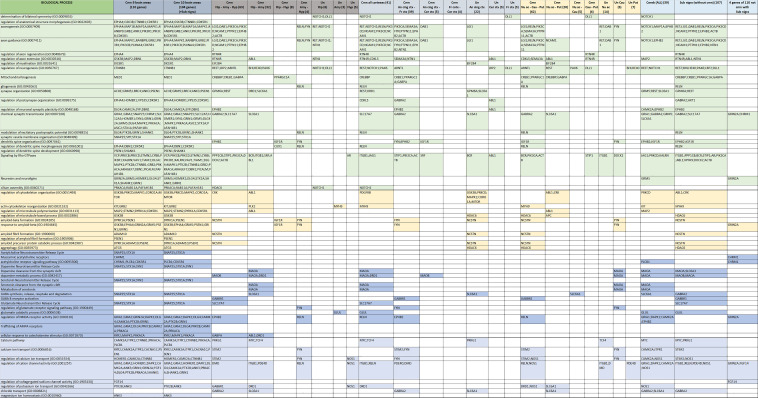
Selected results of biological functions involved in structural development and neurotransmission.

**Figure 7:**
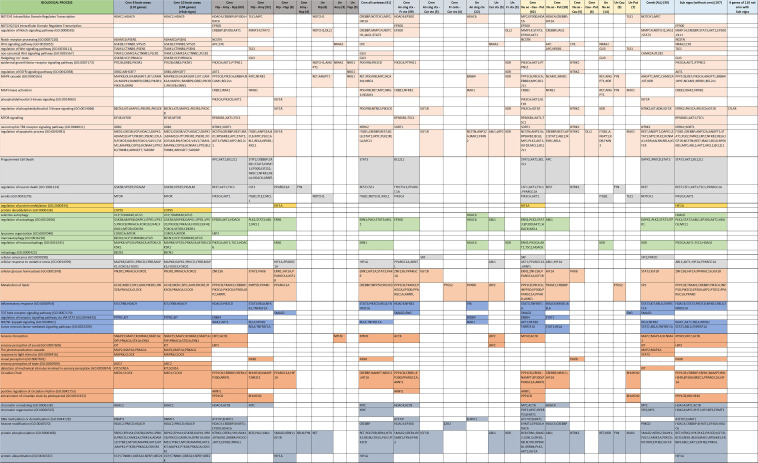
Selected results of biological functions involved in cellular signaling and regulation, metabolism, inflammation, perception and the epigenome.

Identifying the functional role of the genes previously highlighted in our analyses plays a crucial role in understanding the molecular basis of memory degeneration in AD progression. One of the most common hindrances of investigations, such as the one in this work, is the sheer volume of genes and the molecular pathways these involve. Even when clustering the genes by region we ended up with over a thousand results for the GO-BP and Reactome analyses (the full lists for GO-BP and Reactome are presented in multiple color-coded matrices in Supplementary Files S4 and S5, respectively). For this reason, we manually curated the results to identify the most promising ones and cluster them according to their function group. In total, we identified 13 groups of AD genes according to their function across all the AD-involved brain regions. A selection of these results will be presented below and are shown in Figure 6 and Figure 7.

The largest clusters, as expected, involved genes responsible for the anatomical structure development of the nervous system in general. Brain architecture is determined via functions such as the “determination of bilateral symmetry” and “regulation of anatomical structure morphogenesis” which are realized via genes such as *NOTCH1*, *DLL1* and *EPHA4*, *GSK3B*, *CDK5R1*, *CTNNB1*, respectively. In addition, such processes as “Axonogenesis”, “Axon guidance”, the “Regulation of axon regeneration” and the “Regulation of axon extension” are highlighted by some of the same genes, but also *GS3B*, *FYN*, *RELN*, *SYN1*, *DLG4*, *ASIC2*, *GRIN1*, *MCAM*, *PIK3CA*, *PLXNA4*, *LGL1* and *LINGO1*. In addition, the “Regulation of Myelination”, the “Regulation of neurogenesis”, “Mitochondrial biogenesis” and “Gliogenesis” are important components in brain function and can signify, per tissue cell-type, expression risk for AD pathogenesis. Synapse formation, structure and function is brought to the foreground via processes such as “Synapse organisation”, the “Regulation of post-synapse organisation”, the “Regulation of neuronal synaptic plasticity”, “Chemical synaptic transmission”, the “Modulation of excitatory post synaptic potential”, and “Synaptic vesicle membrane organisation”. Additionally, dendritic spines, which are anatomical extensions of neurons, enable synaptic transmission and are facilitated by “Dendritic spine organisation”, the “Regulation of dendritic spine morphogenesis”, and the “Regulation of dendritic spine development”. Furthermore, pathways, such as “Signaling by Rho GTPases”, “Neurexins and neuroligins” and “cilium assembly”, are crucial in the regulation of synaptic cell adhesion and other structural mechanisms.

The histological hallmarks of AD are the anatomical alterations of neurofibrillary tangles, composed of hyperphosphorylated tau protein and amyloid plaque development via extracellular deposits of Aβ (Amyloid beta). These are featured in our results by functions such as the “Regulation of cytoskeleton organisation”, “Actin cytoskeleton reorganisation”, the “Regulation of microtubule polymerisation”, the “Regulation microtubules-based process” and “Amyloid-beta formation”, the “Response to amyloid-beta”, “Amyloid fibril formation, the “Regulation of amyloid fibril formation”, the “Amyloid precursor protein catabolic process”, and “Aggrephagy”.

Another closely related cluster was more specific on genes associated with the way neurotransmitters are produced and affect brain function. This includes the “Acetylcholine neurotransmitter release cycle”, “Muscarinic acetylcholine receptors”, the “Acetylcholine receptor signaling pathway”, the “Dopamine neurotransmitter release cycle”, the “Dopamine clearance from synaptic cleft”, the “Dopamine metabolic process”, the “Serotonin neurotransmitter release cycle”, the “Serotonin clearance from synaptic cleft”, the “Metabolism of serotonin” (*MAOA*), “GABA synthesis, release, reuptake and degradation”, “GABA B receptor activation”, the “Glutamate neurotransmitter release cycle”, the “Regulation of glutamate receptor signaling pathway”, the “Glutamate catabolic process”, the “Regulation of NMDA receptor activity”, “Trafficking of AMPA receptors” and the “Cellular response to catecholamine stimulus”.

In addition, one of the clusters is dedicated to ion channels and ion transport specifically. With such processes as the “Calcium pathway”, “Calcium ion transport”, “Regulation of calcium ion transport”, “Regulation of cation channel activity”, “Regulation of voltage-gated sodium channel activity”, “Regulation of potassium ion transport”, “Chloride transport”, and “Magnesium ion homeostasis”, we highlight their role in ion regulation and homeostasis, which is known to be important in learning and memory-related functions.

These finding are also mirrored in the largest cluster of “signaling” which encases genes associated with neuron migration and the development of neuron projections. Both Reactome and GO-BP allowed us to distinguish and accentuate developmental pathways important in AD pathogenesis, which also include genes from MADgs. The NOTCH pathway, central in developmental processes, is represented by the “NOTCH1/2/3/4 intracellular domain regulates transcription”, the “Regulation of NOTCH signaling pathway, and “NOTCH receptor processing”. The “Wnt signaling pathway”, the “Regulation of Wnt signaling pathway”, and the “Non-canonical Wnt signaling pathway” are important processes in neural progenitor cell differentiation, axon guidance and neuronal plasticity, affecting neuronal physiology in general. The Hedgehog signaling pathway (“Hedgehog ‘on’ state”) also affects neurogenesis, synaptic plasticity and apoptosis, all important factors in AD progression and memory decline. Finally, among the aforementioned, the EGFR pathway also plays the same roles and can be found in the “Epidermal growth factor receptor signaling pathway” and the “Regulation of EGFR signaling pathway”. Interestingly, these signaling pathways extend beyond the development process into late life neurodegeneration, as well as memory.

Independent of neural function and evolvement, several systemic mechanisms are featured in our results and clustered together. Cellular processes, such as cell adhesion, polarity, and survival, and metabolism pathways, such as ERK/MAPK (“MAPK cascade”, “MAP kinase activation”), PI3K/AKT (“phosphatidylinositol 3-kinase signaling”, “regulation of phosphatidylinositol 3-kinase signaling”) and mTOR (“MTOR signaling”), appear to be able to regulate, through their functionality, the memory processes in AD. Activation of the “Neurotrophin TRK receptor signaling pathway” promotes survival and other functional regulations of neuron cells. Excessive apoptosis of neurons is also a staple of neurodegenerative disorders, highlighted in our results by such processes as the “Regulation of apoptotic process”, “Programmed cell death” and the “Regulation of neuron death”. “Anoikis” is also a form of programmed cell death. Over-activation of protein neddylation pathways leads to apoptosis and/or autophagy of neurons (“Regulation of protein neddylation”, “Protein deneddylation”). Moreover, “Selective Autophagy”, “Mitophagy” and “Macroautophagy” are degradative pathways for recycling proteins in cells, and are accompanied by their regulatory processes, such as the “Regulation of autophagy”, “Regulation of macroautophagy” and “Lysosome organization”. Cell aging is also vital to the degeneration component of AD and the atrophy is related to “cellular senescence”. The oxidative stress functionality comes forth in processes like “Cellular response to oxidative stress” and is a key player in cell damage as a defensive factor. “Cellular glucose homeostasis” and “Metabolism of lipids” provide information on the metabolic background of maintaining neuronal function. In addition, inflammatory processes are also prominent in our results with TGF-β, JAK/STAT, NF-κB, TNFα pathway involvement (“TGF beta receptor signaling pathway”, “regulation of receptor signaling pathway via JAK-STAT”, “NIK/NF-kappaB signaling”, “tumor necrosis factor-mediated signaling pathway”), all significant signs of neuroinflammation and immunity activation.

Hypersensitivity to sensory stimuli and perception deficits are known to occur during AD. Several pathways in our results point to senses also influencing memory, with the genes of MADgs highlighting processes like “*Sensory perception*”, “*Sensory processing of sound*”, “Phototransduction cascade”, “Response to light stimulus”, “Visual perception”, “Sensory perception of taste” and “Detection of mechanical stimuli involved in sensory perception”. Furthermore, this hypersensitivity and other functions, for example, learning, are known to be affected by changes in the circadian rhythm and sleep dysregulation which are also notable in the functional role of genes like *MED1*, *CLOCK* (“Circadian clock”), *ARNTL* (“Positive regulation of circadian rhythm”), *PPP1CB*, and *BHLHE40 (*“Entrainment of circadian clock by photoperiod”).

Finally, epigenetic alterations, which are prominent in several pathogenetic mechanisms of disorders, are also featured in our results with processes like “Chromatin remodelling”, “Chromatin organisation”, “DNA methylation or demethylation”, “Histone modification”, “Protein phosphorylation” and “Protein ubiquitination”, bringing them to the foreground. Chromatin regulatory mechanisms crucially affect various stages of neural physiology, neuroplasticity, memory and learning.

As a more general observation, a variety of genes from MADgs appear to have a pleiotropic role in various biological processes in the same brain region, e.g., *NOTCH1* is involved in “Bilateral symmetry”, “Neurogenesis”, “Axonogenesis”, and many more in the Hippocampus. Additionally, the same biological process can be found in multiple brain regions with the same gene involvement, e.g., “Axon guidance” contains *EPHA4* across all studied brain regions.

## 4. Discussion

In this work we identified genes in the literature that are known to be involved in memory mechanisms, as well as genes perturbed during Alzheimer’s Disease. Their intersection (called the Memory in the AD gene set—MADgs) allowed us to study their functional role in AD’s memory deficits. Our results suggest that the pathophysiological heterogeneity of memory is matched by the diversity of genetic and functional insults associated with AD. We found that the distribution of the genes had diverse co-expression profiles across 11 brain regions and are, therefore, likely to be important in multiple stages of synaptogenesis, synaptic plasticity, synaptic transmission, and brain function. In addition, the implicated genes are also possibly co-expressed across multiple cell types, such as neuroblasts, progenitor cells, astrocytes, oligodendrocytes, glia cells and, most noticeably, neurons, of these brain regions. These cell-type specific functions are important indicators of pathological traits [45], and their transcriptomic profiling can lead to better understanding their role in AD symptomology and, more specifically, memory impairment. For this reason, current research includes single-cell RNA sequencing (scRNA-seq), transcriptomics, highlighting the role of gene expression in specific cell types [46,47,48,49]. Akin to scRNA-seq, spatial transcriptomics, a methodology dedicated to assigning cell specific expression profiles to locations on the appropriate histological sections [50], has recently allowed for the identification of specific genes in microglia, astroglia [51] and neurons [52] that can potentially be studied as precursors of AD physiopathology (e.g., the remodeling process of ECM is based on the functional impact of brain-resident macrophages, known as microglia, which are the dominant immune cell in the brain parenchyma. This possible mechanism could help explain how immunity signals can impact the formation of memories). In our approach there seems to be a higher number of MADgs genes involved in regions previously identified for their role in AD onset and progression. For example, 205 out of 265 of the genes of MADgs are co-expressed in the hippocampus and the amygdala, both important in AD activity and memory function. The pleiotropic role of these genes allows for the hypothesis that their perturbation leads to an accumulative cascade effect which might lead, on average, to more severe phenotypes of memory loss. Additionally, neurodevelopment and neurodegeneration pathways seem to share cellular and molecular mechanisms, something noted in the literature and in our results, when studied from the memory impairment perspective. Thus, the memory dysfunction in AD may lie primarily in the degree of the overall functional effect from the specific genes and pathways affected by them, leading to possibly fruitful targets for further investigation.

The interaction between miRNAs and genes has been abundantly established in the current literature regardless of the studied condition. In AD, specifically, several miRNAs have been found potentially important to be characterized as biomarkers of onset and progression [53,54,55]. In this work we take a slightly different approach, identifying miRNAs which interact specifically with our memory related genes in AD. Probably the strongest candidate for further investigation is hsa-miR-16-5p which has been found to interact with 145 out of the 265 genes in our MADgs. hsa-miR-16-5p has been found previously as part of the AD miRNA regulatory network [56], but its role in memory has not been studied yet. On the other hand, miR-124-3p, which interacts with 135 out of the 265 genes in our MADgs, has been previously linked to learning and memory via its interaction with STAT3 and its role in reducing neuroinflammation [57], but also in alleviating neurodegeneration [58]. Additionally, hsa-miR-27a-3p (129/265 MADgs genes) appears to have a neuroprotective function, especially in the hippocampus [59,60], while hsa-miR-34a-5p (121/265 MADgs genes) and mir-155-5p (121/265 MADgs genes) modulate neuroinflammation [61,62], which has been linked multiple times, including in this work, to memory impairment. Most of these miRNAs have been studied in serum/plasma which makes them better candidates as biomarkers than being brain tissue-expressed, but also subtracts from their accuracy and/or importance in AD pathology.

These results point mainly to the dysfunction of memory processes, which is an early-stage effect, rather than the more pronounced memory loss of end-stage AD, often associated more with brain atrophy and cell death. The latter, of course, is also featured through mechanisms like anoikis, autophagy, mitophagy, macrophagy, neddylation, etc., but degeneration effects, especially on the more, apparently, vulnerable component of neurons, the synapses, are over-represented. Looking more closely, during the process of anoikis, integrin-mediated cell attachment to extracellular matrix (ECM) is lost, resulting in a caspase-dependent cell death [63], so a possible overactivation of it might be detrimental to the memory process. In addition, genes in our MADgs provide links to the autophagy lysosomal pathway (ALP) which has been extensively implicated in AD [64,65,66]. As a key mechanism for the degradation of intracellular macromolecules, the ALP can be used to degrade aggregated proteins, the accumulation of which is a characteristic of many adult-onset neurodegenerative diseases, and AD specifically. This leads to intense interest in understanding the role of the ALP since the molecules and mechanisms controlling it are relatively unexplored. In a work by Jegga et al. [67], via a systems biology approach a significant number of genes have been identified for their involvement of ALP, from which 23 are currently highlighted in our own results (*HDAC6*, *MTOR*, *NFKB1*, *ITPR1*, *TSC1*, *LRP2*, *PRKCD*, *SORT1*, *ESR1*, *MAPK8*, *MYC*, *DAPK1*, *TP63*, *MAPK1*, *NRG1*, *ERN1*, *FOXO3*, *ITGB1*, *ATG5*, *AKT1*, *RPS6KB1*, *BECN1*, *BCL2L1*). Furthermore, synaptic plasticity and excitatory transmission are regulated by neuronal neddylation, which contributes to nerve growth, synapse strength, neurotransmission, and synaptic plasticity at normal levels, but the overactivation of protein neddylation pathways leads to apoptosis, neuronal autophagy, and tumor growth [68]. This knowledge enhances our findings that neddylation related genes are highly correlated to memory function in AD.

In general, structural process perturbation appears to be pivotal in memory dysfunction. Adult neurogenesis, especially when disrupted, allows for rapid neurodegeneration [69], and the same has been noted for dendritic spine remodeling [70] and dysfunction of synaptic plasticity [71]. Several other formational processes outlined in our results, such as the cilium assembly [72] and cell adhesion via Rho GTPases [73], are correlated to the hallmarks of AD, Aβ accumulation and Tau misfolding, and their dysfunction can possibly lead to memory impairment. Interestingly, Rho GTPases are already being considered as possible therapeutic targets in AD [74], while the role of cilia, even though under investigation [71], has not yet been utilized therapeutically. The primary cilium is enriched in receptors that mediate the transduction of Hedgehog (Hh), Wnt, Notch, Hippo, G protein-coupled receptors, receptor tyrosine kinases, mTOR, and TGFβ signals [75], all pathways prominent in our results. Disassembly of cilia requires the action of the Aurora A kinase. Aurora A then phosphorylates and stimulates the histone deacetylase HDAC6 [76] (found in our results) which de-acetylates and destabilizes microtubules within the axoneme. Cilia are also important in the regulation of stem cells and regeneration in the adult nervous system [77]. We believe that further experiments to dissect the cellular networks that govern cilium assembly, and to understand the role of cilia in neuronal function are needed to determine if neuronal primary cilia modulate basal hippocampal neuronal activity, allowing them to actively participate in memory formation. These architectural components are also very demanding in energy which can be gained via glucose and lipid metabolism to maintain important functions like neuronal projection and axon myelination [78].

Cell signaling and neurotransmission are highlighted as powerful components of the neurodegeneration–memory complex in our results. Several of the selected signaling pathways, such as Notch, Wnt, Hedgehog and EGFR, are fundamental during development in early life, but are also brought again to the foreground during late life neurodegeneration. Research in AD has focused on acetylcholine as a druggable target [79] of a neurotransmission defect, but in our results, several other neurotransmitters are featured as well, like dopamine, glutamate receptors (NMDA, AMPA), GABA, catecholamine and serotonin. Especially the latter appears to be significant in memory retention [80] of AD and is affected by Tau and Aβ [81]. Additionally, serotonin receptors and neurexins-neuroligins (NRXN-NLGN) interact in various ways, affecting neuronal synapses [82], especially via a molecule which is prominent in our results, the DLG4 [83]. Based on these results, we believe that serotonin, already considered a therapeutic target in depression [84], can be further studied as a target in memory loss and AD.

Aging, several exogenous environmental and epigenetic factors must also be considered when AD and memory dysfunction are studied. Disturbances in the circadian rhythm are already known to affect memory [85,86,87]. Epigenetic mechanisms are critical modulators of synaptic plasticity and memory [88]. Our results indicated that epigenetic regulators such as HDACs, which are established memory-associated genes [89], affect synaptic function, e.g., *HDAC2* is associated with the “regulation of neuron projection development “, and *HDAC4* and *HDAC9* with “inflammatory response”. Vice versa, a transcription factor which affects epigenetic mechanisms like REST, regulates gene expression via epigenetic mechanisms and participates in “synapse organization”. Studying the functional relationship between the epigenomes in AD progression allows us to identify and understand modifications and remodeling of synaptic plasticity involved in the memory process.

The limitations of dedicated bioinformatics approaches are not absent from this work. The validity/importance of information, provided by various expanding and constantly updated databases, is crucial for obtaining the appropriate data for analyses. Hence, the need for better curated and more robust publicly available data constantly arises. In addition, output of computational approaches tends to be bloated, as it can be seen, for example, in our full results of our functional analyses, requiring careful examination and curation in order to produce actionable data, sometimes inadvertently introducing some level of bias. Since most results from bioinformatics pipelines can be considered indications and not proof of how biological systems work, it is important to always complement analyses via different avenues and provide validation in a laboratory setting before claiming importance in clinical practice. On the other hand, computational approaches are crucial, due to the large number of -omics data available to researchers nowadays, and have helped push science to new heights, cutting down money and time costs. To overcome these limitations, future studies could utilize integrated precision-based approaches and perhaps have the ability to process real-time data on living cells under dynamic conditions. This would allow for more powerful analyses of functional genomics and epigenomics.

This work stands as a testament to the complexities and intricacies of the AD-memory complex, but also as a roadmap to several avenues for therapeutic targeting. Our results correlate directly to and are a faithful snapshot of current knowledge, but can also be used for future predictions. We believe that by studying the overall genetic and epigenetic effects on different brain regions during memory decline in AD, we gain a better understanding of how the disease progression affects memory formation and retention, and during which stages this can be therapeutically ameliorated.

## Supplementary Materials

The following supporting information can be downloaded at: https://www.mdpi.com/article/10.3390/biom12020263/s1, Supplementary File S1: Gene lists and PPI network of MADgs; Supplementary File S2: List of genes for each Venn diagram of Figure 5; Supplementary File S3: miRNAs and how many genes of MADgs they affect; Supplementary File S4: GO-BP complete curated and categorized results; Supplementary File S5: Reactome complete curated and categorized results.
